# Research attitudes, practice and literacy among Kenyan palliative care healthcare professionals: an observational, cross-sectional online survey

**DOI:** 10.1186/s12904-022-01091-3

**Published:** 2022-11-24

**Authors:** Powell RA, Ali Z, N Gikaara, S Qanungo, Melikam ES, Cartmell KB

**Affiliations:** 1 Department of Primary Care and Public Health, Faculty of Medicine, Imperial College London, London, England; 2 Ethnicity and Health Unit, NIHR Applied Research Collaboration Northwest London, London, England; 3 MWAPO Health Development Group, Nairobi, Kenya; 4 Kenyan Hospices and Palliative Care Association, Nairobi, Kenya; 5 Department of Medicine, Aga Khan University Hospital, Nairobi, Kenya; 6 College of Nursing, Medical University of South Carolina, Charleston, SC USA; 7 Department of Public Health Sciences, College of Behavioral, Social and Health Sciences, Clemson University, Clemson, SC USA

**Keywords:** Attitudes, Palliative care, Kenya, Africa, Research, Survey, Research capacity

## Abstract

**Background:**

While research is needed to advocate for implementation of global agendas to strengthen palliative care, healthcare professionals’ research literacy must improve to bridge the gap between evidence and practice. A resurgent focus on North-South power disparities, means attention should also focus on understanding low- and middle-income countries’ local agency to implement palliative care research agendas.

**Methods:**

An observational, cross-sectional online survey among Kenyan palliative healthcare professionals currently working at any of the palliative and hospice care organizations operational during January – December 2019, using descriptive statistics.

**Results:**

Among the 93 survey respondents, participants were mainly nurses (50.54%; n = 47). Regarding research attitudes: all agreed/strongly agreed research was important for their professional work. Over nine-tenths (91.21%; n = 83) reported having the skills to conduct research, and 91.30% (n = 84) wanted to conduct research in their clinical work. 90% (90.21%; n = 83) reported supervisory support to conduct research. A comparable proportion (90.22%; n = 83) would undertake research if they could find funding. Regarding research practice: over two-thirds (70.65%; n = 65) reported ever having had a mentor who encouraged them to do research, while approximately half (50.59%; n = 43) reported reading evidence-based journal articles about once per month and attending monthly in-house meetings on palliative care (56.79%; n = 46). Regarding research literacy: while over two-fifths of respondents described their current research literacy level as ‘none’ or ‘beginner’ (44.56%; n = 41), a comparable proportion described it as ‘intermediate’ (45.65%; n = 42), with 9 (9.78%) stating it was ‘advanced’.

**Conclusion:**

The majority of palliative healthcare professionals report having interest, skills and support at work to conduct palliative care research, with a low-to-medium level of research literacy. The current study explored palliative care staff attitudes to, experience in, and literacy with the research process, which is necessary to creating a dialogue on implementing research findings. This study also adds to the global empowerment agenda, addressing inequities in research opportunities and local capacity to own and undertake palliative care research.

**Supplementary Information:**

The online version contains supplementary material available at 10.1186/s12904-022-01091-3.

## Background

While health agendas in low- and middle-income countries (LMICs) are increasingly formulated at the global level (e.g., Universal Health Coverage, Sustainable Development Goals), country-level policy and programmatic operationalisation to ensure supportive legislative, regulatory, financial and programmatic environments, is often premised on successful advocacy by civil society organisations [1]. A key element to the effectiveness of national advocacy is methodologically rigorous, evidence-based research [2], that can inform and embed global agendas to meet localised needs, priorities and preferences [3].

While an internationalist approach—i.e., involving two or more countries, or focused on the global level—to palliative care research is increasingly evident [4], national research endeavours—especially in LMICs—remain relatively scarce. While bibliographic research indicates a significant growth in palliative care research globally—increasing five-fold between 2002 and 2020, with cancer the most popular focus—palliative care research is most commonly conducted in the USA and UK [5], with a body of accumulating research evidence with minimal transferability to LMIC settings. Indeed, research collaborations between LMICs and high-income countries have significant limitations [6]. Not only is there a need to expand palliative care research to non-oncological malignancies, but also for more locally developed and contextualized research [5], especially in LMICs where palliative care service provision and coverage remain relatively under-developed [7, 8] and the need for evidence supporting policy change is imperative. Implementing such research, thereby bridging the gap between evidence and clinical practice effectively and improving the care and treatment provided to patients and their carers, is problematic [9] and premised on a degree of research literacy among those care professionals tasked with that role.

Additionally, the need to establish prioritised palliative care research agendas that are country-level, contextually relevant, acceptable, and receptive to patients’, carers’ and professionals’ needs, pre-dates recent global political agenda shifts [6, 10]. Attention has increasingly focussed on issues of North-South power and resource disparities, and localisation for sustainable development, that are increasingly adopted by non-governmental organisations [11] and major international funders like USAID [12], and in palliative care circles [13–15]. As part of this agenda, there is also a need to explore how global research is ‘owned’ and conducted, including the extent to which and how indigenous palliative care professionals can conduct and lead locally generated research agendas.

Despite advances [16], driven by multiple factors [17], palliative care remains a relatively new discipline across Africa [18], with only six of 53 countries having stand-alone national palliative care policies in 2016 (i.e., Malawi, Mozambique, Rwanda, Swaziland, Tanzania, and Zimbabwe) [19]. In Kenya, which in 2021 released its first ever national palliative care policy [20], the need for effective palliative care is also significant, despite advances in service provision across its 78 public and private hospices and palliative care providers: free-standing hospices (n = 16), hospices and palliative care services in rural areas (n = 3), mission hospital-based hospices and palliative care services (n = 10), government hospitals with palliative care (n = 40), private institutions (n = 9) [21]. Past palliative care research in Africa has often been characterized by short-term, project-specific commitment, inadequate financing, over-dependency upon key individuals, communication difficulties, and an over-emphasis on North-South partnerships inevitably led by Northern partners that fail to leave a historical footprint upon which further research capacity (skills and knowledge) can be built [6].

Indeed, while research in Kenya, and East Africa generally, is relatively advanced, much more needs to be done [22]. There is need, among others, to explore, determine and enable the capacity of indigenous palliative care professionals to understand and take an active part in implementing locally generated research agendas. While some of the contributory factors to that status quo are known [22] —including the absence of palliative care academic pathways in Kenyan medical schools—the extent to which healthcare professionals’ attitudes towards, experience with, and literacy in, the research process, upon which educational interventions could potentially be initiated, has been neglected.

To fill this gap in the literature, this study was designed to explore the attitudes, practices and literacy related to palliative care research among clinical team members working in palliative care and hospice organizations across Kenya. The reported findings are part of a larger study aiming to determine a prioritised Kenyan palliative care research agenda.

## Methods

### Study aim

The aim of this study was to explore the attitudes, practices and literacy related to palliative care research among clinical team members working in palliative care and hospice organizations across Kenya.

### Study design

We used an observational, cross-sectional survey study design.

### Study population

Participants were all Kenyan palliative healthcare professionals (e.g., doctors, nurses, social workers, nutritionists, and any other operational allied professionals) currently working at any of the palliative and hospice care organizations, covering both rural, peri-urban and urban settings, across the country that were operational during January – December, 2019, before ethical permission was granted for the study. Data collection was undertaken in 2020, following that permission.

### Survey procedure and sample size

Palliative healthcare professionals in Kenya were invited to participate in the survey by sending a letter to the Chief Executive Officer/leader of each palliative care and hospice organization in Kenya, asking them to share the survey with all their clinical team members and by invitation through the Kenya Hospices and Palliative Care Association (KEHPCA) member listserv of palliative care and hospice providers in Kenya. Two email reminders were also sent to encourage contacts to distribute the survey to their clinical team and encourage broad survey participation. Respondents had the option to either fill out a hard copy survey and mail it back to our team or to fill out the survey online in Redcap (Research Electronic Data Capture), a HIPAA (Health Insurance Portability and Accountability Act)-compliant, cloud-based survey tool commonly used for research. Because we asked our partners to share the survey with others in their organization, and were unable to obtain definitive staff listings for each service provider, we were unable to obtain the full denominator of operational hospice and palliative care clinical team members in Kenya, and therefore unable to calculate a study response rate.

### Survey instrument

A module of questions on research attitudes, practice and literacy were adopted with permission from an international project promoting research literacy in professional chaplaincy.^23^ The initial survey, which consisted of six items measuring interest and attitudes regarding research and self-rated research literacy, underwent face and content validation.

The survey included socio-demographic (e.g., age, gender, educational level attained) and occupational questions (e.g., job title, length of time in profession and present hospice / palliative care site, training received, expressed as an open-ended question) and 15 questions related to research: ten regarding attitudes to research (six applying to respondents personally, four applying to their profession, i.e., their professional work, clinical work, as a discipline and relating to building positive relationships with others); four regarding research practice; and one regarding their self-perceived level of research literacy (see Appendix 1 for the module questions). The ten attitudinal questions used a 5-point Likert-type format, with agreement with given statements ranging from 1 = Strongly agree to 5 = Strongly disagree, and were supplemented by a few open-ended questions. While not formally further tested directly among Kenyan palliative care professionals, the module of questions was reviewed thoroughly by the authors (which include a Kenyan palliative care clinical doctor [ZA] and research project manager [NG]) to ensure they were comprehendible in a Kenyan context.

### Informed consent

The study questions were preceded by information explaining the study to potential participants, their roles, the fact that it was voluntary, and that they could refuse to participate or withdraw from it at any point. Informed consent for survey respondents was understood to have been given if they completed the questionnaire.

### Inclusion criteria

Participating staff members had to be: (1) adults (i.e., 18 years or older); (2) active hospice or palliative care clinical team members (e.g., doctors, nurses, social workers, nutritionists and any other operational allied professionals); and (3) employed in palliative care in Kenya for at least one year.

### Data management and analysis

#### Data management

As mentioned, some individuals filled out the survey online in Redcap, which was used to manage the survey records, and others mailed in their survey, which was subsequently entered manually into Redcap by the research team. All participants were assigned a unique subject ID number in the software, and data were stored anonymously in the cloud-based database. Data were only accessible to the research team members and to the ethics boards (in Kenya and the USA), as required. Deidentified data were shared with the Kenyan research team through remote access to Redcap.

#### Data analysis

Descriptive statistics were calculated for all variables, including those from the Likert-type questions, with value proportions compared using SPSS (Statistical Package for the Social Sciences) (Version 12.0). Given the limited data variability to enable sub-analysis, bivariate analysis was only conducted for a small number of research-related dependent variables, where the largest proportion response was less than 80.00%, using the Fisher’s exact test.

## Results

### Participant demographics

Among the 93 survey respondents, mean age was 43.47 years (SD + 12.37; range 23–75 years) (data not shown)—most aged under 60 (89.88%; n = 89)—with a greater proportion of females (66.67%; n = 62) and the vast majority having attended university-level education (90.80%; n = 79) (Table 1). Professionally, participants were mainly comprised of nurses (50.54%; n = 47), doctors (10.75%; n = 10) and social workers (9.68%; n = 9). A slightly larger proportion of participants had been in their current profession for 5–10 years (30.00%; n = 27)—with a median of 10 years and a range of 1–50 years (data not shown). Approximately half had worked in their present care site for less than 5 years (52.44%; n = 43), with the largest proportion of sites located in urban settings (46.59%; n = 41), and in lower level categorised service facilities (65.06%; n = 54).

**Table 1:** Sample characteristics: Socio-demographic, occupational and duration of practice

CHARACTERISTICS	FREQUENCY
	**n (%)**
**Age** (N = 89) 20–39 40–59 ≥60	37 (41.57) 43 (48.31) 9 (10.11)
**Gender (N = 93)** Male Female	31 (33.33) 62 (66.67)
**Highest level of education you have received** (N = 87) Primary Secondary University	0 (0.00) 8 (9.20) 79 (90.80)
**Job title (N = 93)** Physician Nurse Social Worker Psychologist Community Health Worker Volunteer Administrator / Manager Health educator Other professional *Clinical Officer* *Nutritionist* *Physiotherapist* *Pharmacist* *Psychological counsellor*	10 (10.75) 47 (50.54) 9 (9.68) 1 (1.08) 1 (1.08) 2 (2.15) 6 (6.45) 3 (3.23) 14 (15.05) *6* *1* *4* *2* *1*
**How long have you been in your current profession in years? (N = 90)** <5 5–10 11–20 21–30 31–40 >40	23 (25.56) 27 (30.00) 18 (20.00) 16 (17.78) 5 (5.55) 1 (1.11)
**Location of hospice/PC site (N = 88)** Urban Peri-urban Rural	41 (46.59) 26 (29.55) 21 (23.86)
Service level of hospice/PC site* (N = 83) Higher level Lower level	29 (34.94) 54 (65.06)
**How long have you worked at your present hospice/PC site in years? (N = 82)** <5 5–10 11–20 >20	43 (52.44) 31 (37.80) 7 (8.54) 1 (1.22)

Note: Categories for Kenyan service facilities range from level 1 (e.g., community health facility) to level 6 (i.e., national referral and teaching hospitals and specialised hospitals). For this table, higher level service providers grouped together level 5 and 6 facilities compared to those from levels 4 and below

The most common types of palliative care training received included a higher diploma in palliative care (27.27%; n = 27), a palliative care certificate (12.12%; n = 12), End-of-Life Nursing Education Consortium training (14.04%; n = 14), a Bachelor’s degree in palliative care (9.09%; n = 9), and a Master’s degree in advanced practice nursing (oncology and palliative care) (8.08%; n = 8) (Table 2).

**Table 2:** Types of palliative care training received by respondents (N = 93)

CHARACTERISTICS	FREQUENCY
	**N (%)**
Higher Diploma in PC	27 (27.27)
Fellowship in PC	1 (1.01)
Bachelors in PC	9 (9.09)
Basic certificate /from KEHPCA	5 (5.05)
Certificate in PC	12 (12.12)
ELNEC/End of Life Nursing Education Consortium	14 (14.04)
4-month oncology course at International Cancer Institute	1 (1.01)
Internship 1 year	1(1.01)
MSc advanced practice nursing (oncology and palliative care)	8 (8.08)
Others^a^	21 (21.21)
TOTAL	99 responses

^**a**^ Others include advocacy for palliative care, counselling, nutrition and palliative and cancer awareness, cancer care and support, guidance on outcome measurements, spirituality, home-based care, introduction to palliative care, legal issues in palliative care, pain management, psychosocial therapy, sexuality in palliative care, palliative care for health professionals and on-the-job training

^b^ The responses for the population of participants who did not complete the questionnaire might exceed N total because some respondents indicated > 1 palliative care training

### Research attitudes

In responding to questions related to their profession, all (n = 93) agreed or strongly agreed palliative care research was important for their professional work, and that research would benefit the field of palliative care as a discipline (n = 93). Most (79.35%; n = 73) thought research was highly valued at the facility they worked, but over two-thirds (68.48%; n = 63) felt research-based activities get in the way of building positive relationships with others (Table 3). The attitude that research negatively impacted positive relationships was less commonly endorsed by physicians, as compared to nurses and other healthcare workers (P = < 0.0001), and more commonly endorsed by those working in a non-urban setting (P = 0.0022) and lower service level facility (P = 0.0281).

**Table 3:** Research engagement among respondents

Research engagement	Participants (n; %) (N = 93)
**Research in palliative care is very important to my professional work** (N = 93) Strongly Agree/Agree No opinion Strongly Disagree/Disagree	93 (100.00) 0 (0.0) 0 (0.0)
**As a result of my palliative care education experience, I have the skills to conduct research** (N = 91) Strongly Agree/Agree No opinion Strongly Disagree/Disagree	83 (91.21) 3 (3.30) 5 (5.50)
**I can explain the difference between quantitative and qualitative research** (N = 93) Strongly Agree/Agree No opinion Strongly Disagree/Disagree	86 (92.47) 5 (5.38) 2 (2.16)
**I would like to conduct research on some aspect of my clinical work** (N = 92) Strongly Agree/Agree No opinion Strongly Disagree/Disagree	84 (91.30) 7 (7.61) 1 (1.09)
**Research is highly valued at the facility where I work** (N = 92) Strongly Agree/Agree No opinion Strongly Disagree/Disagree	73 (79.35) 11 (11.96) 8 (8.69)
**Palliative care as a discipline will benefit from research** (N = 93) Strongly Agree/Agree No opinion Strongly Disagree/Disagree	93 (100.00) 0 (0.0) 0 (0.0)
**I feel that research-based initiatives in palliative care get in the way of building positive relationships with others** (N = 92) Strongly Agree/Agree No opinion Strongly Disagree/Disagree	63 (68.48) 6 (6.52) 23 (25.00)
**I would do research but cannot find the extra time in my schedule for it** (N = 92) Strongly Agree/Agree No opinion Strongly Disagree/Disagree	50 (54.35) 6 (6.52) 36 (39.13)
**My supervisor would encourage me to do research if I pursued it** (N = 92) Strongly Agree/Agree No opinion Strongly Disagree/Disagree	83 (90.21) 8 (8.70) 1 (1.09)
**I would do research if I could find funding for it** (N = 92) Strongly Agree/Agree No opinion Strongly Disagree/Disagree	83 (90.22) 8 (8.70) 1 (1.09)
**Did you ever have a mentor who encouraged you to do research?** (N = 92) Yes No	65 (70.65) 27 (29.35)
**How often do you read evidence-based journal articles?** (N = 85) Never About once a month 2–3 times a month Weekly	9 (10.59) 43 (50.59) 22 (25.88) 11 (12.94)
**How often do you attend structured in-house meetings to learn more about palliative care / hospice topics?** (N = 81) Never About once a month 2–3 times a month Weekly	9 (11.11) 46 (56.79) 12 (14.81) 14 (17.28)
**I have completed a master’s thesis or doctoral project that required original research** (N = 92) Yes No	14 (15.22) 78 (84.78)
**How would you describe your present level of research literacy?** (N = 92) None Beginner Intermediate Advanced	5 (5.43) 36 (39.13) 42 (45.65) 9 (9.78)

In more personal terms, 91.21% (n = 83) reported having the skills to conduct research as a result of their palliative care education experience, 92.47% (n = 86) felt they could explain the difference between quantitative and qualitative research, and 91.30% (n = 84) wanted to conduct research on some aspect of their clinical work. Moreover, 90.21% (n = 83) reported their supervisor would encourage them to do research they might pursue, and a comparable proportion (90.22%; n = 83) would undertake research if they could find funding, but over half (54.35%; n = 50) said they could not find the extra time needed to do research, significantly associated with those working in non-urban areas (P = 0.0003). The lack of extra time was especially endorsed by physicians compared to nurses and other healthcare workers (P = < 0.05), and those in higher level facilities (P = 0.0388).

### Research practice

Over two-thirds (70.65%; n = 65) of respondents reported ever having a mentor who encouraged them to do research and was significantly associated with not being in the nursing profession (P = 0.0172) and working in an urban setting (P = 0.0143). Most (89.41%; n =76) reported reading evidence-based journal articles, about once per month, with physicians (P = 0.0002) and those working in higher level facilities (P = 0.0051) reading more frequently. Most (88.89%, n = 72) reported attending monthly structured, in-house meetings on palliative care, while 84.78% (n = 78) had not completed an advanced higher education dissertation requiring original research.

### Research literacy

Over two-fifths of respondents described their current research literacy level as ‘none’ or ‘beginner’ (44.56%; n = 41), and a comparable proportion described it as ‘intermediate’ (45.65%; n = 42), with 9 (9.78%) stating it was ‘advanced,’ with physicians proportionately considering themselves more advanced than nurses and other healthcare workers (P = < 0.0001). A statistically significant association was also found between working in a more urban setting and a self-perceived more advanced level of research literacy (P = < 0.0001).

### Site research activity

While over half of respondents stated their site conducts regular service audits (51.06%; n = 24) and were keen to participate in future palliative care research (93.48%; n = 43), the vast majority of sites had not been actually involved in palliative care research (73.33%; n = 33) and had overwhelmingly not published palliative care research in academic journals (91.30%; n = 42) (table not shown).

Participants were also asked about what types of research had been conducted within their palliative care and hospice organisations. Most commonly, respondents reported carrying out descriptive studies (e.g., cancer situation analyses, statistical profiles, cancer prevalence studies, studies of factors influencing quality of home care, and factors influencing early integration of palliative care services). One respondent reported helping conduct a randomized controlled trial, and several reported carrying out qualitative and/or mixed methods research.

When asked about specific barriers to conducting future research at their respective sites, most (n = 19) reported lack of funding, lack of research training among staff (n = 12), and the length of wait time to get research underway (n = 10). Less frequently mentioned barriers were: lack of resources (e.g., reference materials [journals, books, previous research papers], data software, computers and accessories (n = 7), poor health record system / cancer registries for obtaining data (n = 3), and high staff workload (n = 2), as well as lack of government support, facility size, lack of facility resources for research, lack of transportation, no forum nationally to discuss research, the lack of recognition of palliative care within their facility, and the tedious process of obtaining research approvals.

Individual barriers to conducting research were reported as poor clinical research skills (n = 4), the lack of mentorship, supervision or guidance (n = 3), stigmatization / discrimination, lack of motivation and possible fear of being exposed to COVID-19 during hospital visits. External barriers to research were reported as lack of interest from patients and community members (n = 4), attitudes to research (n = 2), and inadequate sample size / death of patients given their late referral to the services, lack of cooperation from community members, lack of incentives for study participants and community health workers, changing trends in the field of research (e.g., technology), reluctance from community members (i.e., cultural restrictions), and COVID-19 restrictions.

## Discussion

Our study had limitations, foremost among them being the relatively small sample size. Unlike Snowden et al. [23], who secured a sample size of 2,092 chaplains in their study of research attitudes and a response rate of 14%, our sample size was much smaller, and it proved impossible to calculate the response rate. Secondly, there was potential for self-selection bias among respondents who chose to answer the research module of questions [24], which could limit the generalizability of the results to the broader population of palliative and hospice care providers in Kenya. Thirdly, the study findings could have been enhanced by more in-depth qualitative research; this is an area that the authors intend pursuing in the future. Despite these limitations, our findings contribute to contemporary discussions of the role of research in palliative care settings.

For several years, advocates have demanded increased attention to palliative care research in LMICs generally [25], research that is contextually appropriate and valid, that could be a fifth pillar—along with policy, education, drug availability, and implementation—in the World Health Organization’s public health model [26]. While research in LMIC settings generally is increasing, Potts et al. [27] recently found that only limited high-quality evidence from low-resource countries is available to document intervention outcomes and called for rigorous experimental studies and greater measurement of multidimensional aspects of palliative care to advance palliative carein such locales.

In Africa, similar calls have been made to build an evidence base to stimulate and support service development [9, 28, 29], focusing especially on the generation of patient-reported outcome measures [30–32] and building a prioritised research agenda [33].

However, developing a localised evidence base to advance service development and improve patient welfare is redundant if the evidence base itself is not understood by those frontline care clinicians and staff for whom it is intended and who deliver care. A precursor to developing a critical cadre of indigenous palliative healthcare professionals that can help implement research findings and advance a locally generated research agenda, is an understanding of their attitudes to, experience of, and literacy in the research process. This study sought to do that and found reassuring results upon which future strategic education and research can build.

Encouragingly, our results indicate there is a receptive ground for research among Kenyan palliative care professionals, with people eager to participate actively in the process rather than being its passive recipients, facilitators or data collectors. Not only did the overwhelming majority perceive palliative care research as important to their profession, but they also thought it was highly valued at the facility they worked, and the vast majority reported having the skills necessary to conduct that research. Whilst we cannot determine what exactly is interpreted by respondents by the term “conduct research”—i.e., confident enough to develop and implement research projects or simply facilitate research driven by others—it does suggest participants believe they have some level of skills that would contribute to implementing research. This wider finding mirrors results from the limited number of similar studies exploring research competency among professional groups. Snowden et al. [23] found that among international chaplains, 70% believed in the importance of research literacy, and 81% believed in the value of research within the profession.

However, clear challenges and barriers exist to participating in research work for Kenyan palliative healthcare professionals. Important among these is available time, a personal barrier to engaging in research reported among other clinical workers in LMICs and high-income countries [34, 35]. Similarly, many respondents stated research activity can impact upon building positive relationships with others, which we speculate could mean taking time away from patient care, challenging trust with patients and /or families involved in research and applying pressure to healthcare peers to find time to undertake the research. Important variations exist, however; physicians reported positive relationships as less problematic than other professionals, which may be associated with reduced direct patient and family contact time. Similarly, differences existed between those working in urban compared to non-urban areas, and those working in higher service level facilities. It can be reasonably speculated that the resources and infrastructure (e.g., access to journals, mentors) needed to facilitate a conducive research culture are more likely to be present in such settings, while physicians reporting insufficient time to undertake research potentially associated with a greater willingness to conduct research compared to those in other professions and settings.

There is a need in this respect, especially in more rual areas, for time-sensitive, tiered opportunities for research understanding and work, and for engaging with in-country centres of higher education to facilitate research openings, as well as fostering journal clubs and enabling subscription to academic journals. Researchers working with Kenyan palliative care staff could embed specific actions within their projects (e.g., educational opportunities through research-based learning in practice) and create more structured educational opportunities in established research programmes, contributing to a facilitative and participatory learning environment and culture within clinical care sites.

## Conclusion

Our work appears to be the first study to explore palliative care staff attitudes to, experience in, and literacy with the research process, creating a much-needed dialogue on facilitating research literacy to implement research findings. It also adds to the global empowerment agenda, seeking to address inequities in research opportunities and by seeking to build meaningful local capacity to own and undertake palliative care research within those LMICs expected to implement the results into care provision.

## Electronic supplementary material

Below is the link to the electronic supplementary material.

Supplementary Material 1

## Data Availability

The datasets generated and/or analyzed during the current study are not publicly available because we are still using the dataset to write another primary paper from other domains on the survey, but will be made available from the corresponding author on reasonable request once our primary papers are published.

## Declarations

### Disclaimer

This article presents independent research. The views expressed in this publication by Richard A. Powell (RAP) are not necessarily those of the NIHCR or the Department of Health and Social Care, London, UK.

## List of abbreviations

HIPAA: Health Insurance Portability and Accountability Act
KEHPCA: Kenya Hospices and Palliative Care Association
LMIC: Low- and middle-income country
Redcap: Research Electronic Data Capture
SPSS: Statistical Package for the Social Sciences

## References

[1] Chapman S (2004). Advocacy for public health: A primer. J Epidemiol Community Health.

[2] Mayne R, Green D, Guijt I, Walsh M, English R, Cairney P (2018). Using evidence to influence policy: Oxfam’s experience. Palgrave Commun.

[3] Mwangi-Powell FN, Debere S, Powell RA, Baguma A (2011). Successful advocacy for palliative care: a toolkit.

[4] Clark J, Gardiner C, Barnes A (2018). International palliative care research in the context of global development: a systematic mapping review. BMJ Support Palliat Care.

[5] Abu-Odah H, Molassiotis A, Liu JYW. Global palliative care research (2002–2020): bibliometric review and mapping analysis. BMJ Support Palliat Care Published Online First: 09 August 2021. doi: 10.1136/bmjspcare-2021-002982. 10.1136/bmjspcare-2021-002982 PMC9691821 34373283

[6] Powell RA, Harding R, Namisango E, Katabira E, Gwyther L, Radbruch L (2013). Palliative care research in Africa: An overview. Eur J Pall Care.

[7] Clark D, Baur N, Clelland D, Garralda E, López-Fidalgo J, Connor S (2020). Mapping levels of palliative care development in 198 countries: The situation in 2017. J Pain Symptom Manage.

[8] Kebudi R, Cakir FB, Silbermann M. Palliative care in high and low resource countries. Curr Pediatr Rev. 2021; Apr 5. doi: 10.2174/1573396317666210405143649. Online ahead of print. 10.2174/1573396317666210405143649 33820519

[9] Kristensen N, Nymann C, Konradsen H (2016). Implementing research results in clinical practice - the experiences of healthcare professionals. BMC Health Serv Res.

[10] Selman LE, Brighton LJ, Sinclair S, Karvinen I, Egan R, Speck P (2018). Patients’ and caregivers’ needs, experiences, preferences and research priorities in spiritual care: A focus group study across nine countries. Palliat Med.

[11] Mathews D. Dear USAID; let’s make sure that “local” really means “local”. BOND, 11 November, 2021. https://www.bond.org.uk/news/2021/11/dear-usaid-lets-make-sure-that-local-really-means-local. Accessed 23 Nov 2021.

[12] Power S. A new vision for global development. 4 November, 2021, Georgetown University. https://www.youtube.com/watch?v=2A179iUzc9w&t=2s. Accessed 23 Nov 2021.

[13] Burt J (2011). Back to basics: researching equity in palliative care. Palliat Med.

[14] Editorial (2015). A call for equity in the delivery of UK palliative care. Lancet.

[15] Gott M, Stajduhar K. Equity-oriented palliative care. European Association of Palliative Care. 22 November, 2021. https://eapcnet.wordpress.com/2021/11/22/equity-oriented-palliative-care-submit-to-the-palliative-medicine-special-issue/. Accessed 23 Nov 2021.

[16] Powell RA, Mwangi-Powell FN, Kiyange F, Radbruch L, Harding R (2011). Palliative care development in Africa: How can we provide enough quality care?. BMJ Support Palliat Care.

[17] Rhee JY, Garralda E, Namisango E, Luyirika E, de Lima L, Powell RA, et al. Factors affecting palliative care development in Africa: In-country experts’ perceptions in seven countries. J Pain Symptom Manage. 2018 Feb 2. pii: S0885-3924(18)30029-0. doi: 10.1016/j.jpainsymman.2018.01.009. [Epub ahead of print]. 10.1016/j.jpainsymman.2018.01.009 29409870

[18] Rhee JY, Garralda E, Torrado C, Blanco S, Ayala I, Namisango E (2017). Palliative care in Africa: A scoping review from 2005-16. Lancet Oncol.

[19] Emmanuel BK, Luyirika EBK, Namisango E, Garanganga E, Monjane L, Ginindza N (2016). Best practices in developing a national palliative care policy in resource limited settings: lessons from five African countries. Ecancermedicalscience.

[20] Ministry of Health (2021). Kenya Palliative Care Policy, 2021–2030.

[21] Kenya Hospices and Palliative Care Association. Hospices and palliative care units in Kenya. Kenya Hospices and Palliative Care Association. https://kehpca.org/pc-providers/. Accessed 14 Oct 2021.

[22] Namisango E, Powell RA, Kariuki H, Harding R, Luyirika E, Mwangi-Powell FN (2013). Palliative care research in Eastern Africa. Eur J Pall Care.

[23] Snowden A, Fitchett G, Grossoehme DH, Handzo G, Kelly E, King SDW (2017). International study of chaplains’ attitudes about research. J Health Care Chaplain.

[24] McGrath RE, Mitchell M, Kim BH, Hough L (2010). Evidence for response bias as a source of error variance in applied assessment. Psychol Bull.

[25] Anon (2006). Adoption of a declaration to develop a global palliative care research initiative. Prog Palliat Care.

[26] Harding R, Selman L, Powell RA, Namisango E, Downing J, Merriman A (2013). Research into palliative care in sub-Saharan Africa. Lancet Oncol.

[27] Potts M, Cartmell KB, Nemeth L, Bhattacharjee G, Qanungo S (2018). A systematic review of palliative care intervention outcomes and outcome measures in low-resource countries. J Pain Symptom Manage.

[28] Harding R, Powell RA, Downing J, Connor S, Mwangi-Powell F, Defilippi K (2008). Generating an African palliative care evidence base: The context, need, challenges and strategies. J Pain Symptom Manage.

[29] Powell RA, Downing J, Radbruch L, Mwangi-Powell FN, Harding R (2008). Advancing palliative care research in Africa: From Venice to Nairobi and beyond. Palliat Med.

[30] Downing J, Ojing M, Powell RA, Ali Z, Marston J, Meiring M (2012). A palliative care outcome measure for children in sub-Saharan Africa: Early development findings. Eur J Palliat Care.

[31] Harding R, Mwangi-Powell FN, Gwyther L, Dinat N, Powell RA (2010). Brief Global Reports – How can we improve palliative care patient outcomes in low and middle income countries? Successful research approaches in sub-Saharan Africa. J Pain Symptom Manage.

[32] Powell RA, Downing J, Harding R, Mwangi-Powell F, Connor S, on behalf of the APCA M&E Group (2007). Development of the APCA African Palliative Outcome Scale. J Pain Symptom Manage.

[33] Powell RA, Harding R, Namisango E, Katabira E, Gwyther L, Radbruch L (2013). Palliative care research in Africa: Consensus building for a prioritised agenda. J Pain Symptom Manage.

[34] Ataee M, Hesamzadeh A, Kheradmand M (2015). Research barriers from experts’ viewpoints who attended the research workshops of Mazandaran University of Medical Sciences. J Med Life.

[35] Royal College of Physicians (2016). Research for all: Building a research-active medical workforce.

