# Supplementary material for: Research attitudes, practice and literacy among Kenyan palliative care healthcare professionals: an observational, cross-sectional online survey
Source: BMC Palliat Care. 2022 Nov 24;21:206. doi: 10.1186/s12904-022-01091-3 (PMC9684766; doi:10.1186/s12904-022-01091-3)
Supplement: Supplementary file 1 — Supplementary Material 1 [file 12904_2022_1091_MOESM1_ESM.docx]

**Supplemental File 1: Survey items used to assess palliative care staff research**

**attitudes, practice and literacy**

1. **Attitudes to research**

Please respond to the following statements using the following five-point scale:

**1. Strongly agree 2. Agree 3. No opinion 4. Disagree 5. Strongly disagree**

*Research in palliative care is very important to my professional work.*

**1. Strongly agree 2. Agree 3. No opinion 4. Disagree 5. Strongly disagree**

*As a result of my palliative care education experience, I have the skills to conduct research.*

**1. Strongly agree 2. Agree 3. No opinion 4. Disagree 5. Strongly disagree**

*I can explain the difference between quantitative and qualitative research.*

**1. Strongly agree 2. Agree 3. No opinion 4. Disagree 5. Strongly disagree**

*I would like to conduct research on some aspect of my clinical work.*

**1. Strongly agree 2. Agree 3. No opinion 4. Disagree 5. Strongly disagree**

*Research is highly valued at the facility where I work.*

**1. Strongly agree 2. Agree 3. No opinion 4. Disagree 5. Strongly disagree**

*Palliative care as a discipline will benefit from research.*

**1. Strongly agree 2. Agree 3. No opinion 4. Disagree 5. Strongly disagree**

*I feel that research-based initiatives in palliative care get in the way of building positive relationships with others.*

**1. Strongly agree 2. Agree 3. No opinion 4. Disagree 5. Strongly disagree**

*I would do research but cannot find the extra time in my schedule for it.*

**1. Strongly agree 2. Agree 3. No opinion 4. Disagree 5. Strongly disagree**

*My supervisor would encourage me to do research if I pursued it.*

**1. Strongly agree 2. Agree 3. No opinion 4. Disagree 5. Strongly disagree**

*I would do research if I could find funding for it.*

**1. Strongly agree 2. Agree 3. No opinion 4. Disagree 5. Strongly disagree**

1. **Research practice**

*Did you ever have a mentor who encouraged you to do research?*

Yes No

*How often do you read evidence-based journal articles?*

**1. Never 2. About once / month 3. 2-3 times / month 4. Weekly**

*How often do you attend educational grand rounds?*

**1. Never 2. About once / month 3. 2-3 times / month 4. Weekly**

*I have completed a master’s thesis or doctoral project that required original research.*

Yes No

1. **Research literacy**

*How would you describe your present level of research literacy?*

None (It is difficult for me to understand any research papers or presentations)

Beginner (I can understand the basic points in simple research papers or presentations)

Intermediate (I understand differences between several types of study designs and have a basic grasp of the most commonly reported statistical tests)

Advanced (I am able to describe knowledgably the strengths and weaknesses of some of the research reported in articles I read or presentations I hear.)
